# A call to give a voice to people with intellectual disabilities in Africa through inclusive research

**DOI:** 10.4102/ajod.v12i0.1127

**Published:** 2023-04-25

**Authors:** Callista K. Kahonde

**Affiliations:** 1 Department of Global Health, Faculty of Medicine and Health Sciences, Stellenbosch University, Cape Town, South Africa

**Keywords:** intellectual disabilities, inclusive research, Africa, inclusion, human rights

## Abstract

Research looking into the day-to-day lives of people with intellectual disabilities (ID) is on the increase in Africa. However, not enough is being done to include people with ID as active contributors to this research through inclusive approaches. Inclusive research empowers people with ID as they have the agency and autonomy to speak for themselves and they are given an active voice in the research process and outcomes. This leads to services that cater for what matters to people with ID themselves as opposed to having their needs defined by other people. The common myths and misconceptions attached to ID in Africa, which increase stigma towards people affected by this type of disability can be abated by their visibility in research and evidence of their ability to express themselves. This article makes a call to researchers on the African continent to include people with ID in research as active contributors to the research and not simply as research subjects or respondents. A background is given of global developments that have occurred in inclusive research based on the literature and the author’s personal experience, which African researchers can learn from while taking cognizance of the specific needs of their own contexts. This is followed by highlighting the gaps in Africa. The article ends with a discussion of possible reasons for a lack of inclusive research in Africa and suggestions and recommendations to address this gap.

## Introduction

Traditionally, people with intellectual disabilities (IDs) were victims of extreme marginalisation, stigma, discrimination and other exclusionary practices (Scior 2016). There have been notable efforts and initiatives in recognition of their right to social participation and inclusion globally, for example, through the normalisation model that was initially practiced in the Scandinavian countries and also had a strong influence in the United States of America (USA), Australia and Europe (Culham & Nind 2003). Deinstitutionalisation (Mansell & Beadle-Brown 2010) and the emergence of policies and programmes informed by the social model of disability and the United Nations Convention on the Rights of Persons with Disabilities (UNCRPDs) (United Nations 2006) have also contributed to promoting their human rights and social inclusion. Although there was a lack of documentation on the lives of people with ID in Africa in the past, global improvements, especially the advent of the social model and the UNCRPD have resulted in recognition of the rights of this group within the continent across different sectors of society. However, a lot still needs to be done for many of them to experience all life domains on par with their contemporaries without ID. This applies to their meaningful inclusion in research that seeks to understand issues impacting on their lives (Capri & Coetzee 2012). People with ID can be recruited in research as participants, or they can be involved as co-researchers who contribute to the data collection and analysis process or research can even be more inclusive by including them in all the stages of research from initiating, planning, executing and guiding the process (Bigby, Frawley & Ramcharan 2014).

The exclusion of people with ID as active participants in research projects is a universal phenomenon although significant strides have been made in promoting and conducting inclusive research in high-income countries, particularly Australia, the United Kingdom, Ireland and the Netherlands (Bigby et al. 2014; Strnadová et al. 2015; Tilley et al. 2021; Walmsley 2001). Since the turn of the 21st century, there has been a proliferation of published original inclusive research and other writings on inclusive practices. Walmsley (2001) and Johnson and Walmsley (2003) are the two widely quoted seminal literature sources discussing and defining the concept of inclusive research. Walmsley (2001:188) defines inclusive research as research in which people with ID are involved as active contributors to the research and not simply as ‘research subjects or respondents’. This entails research ‘with’ people with ID that is inclusive versus research ‘about’ them (Bigby et al. 2014; Walmsley 2001). This research does not only empower people with ID through the research process, but it leads to outcomes that embody their voice and desires as they actively partake in the research as collaborators, advisors, leaders or controllers of the research (Bigby et al. 2014; Johnson & Walmsley 2003). It is the kind of research exemplifying the *Nothing about us without us* principle (Charlton 1998) and leads to evidence-informed services that cater for what matters to people with ID themselves as opposed to having their needs defined by service providers, families and policy makers.

There have been other prominent developments in inclusive research with people with ID on a global level, for example, the launch of the Guidelines for Co-Producing Research with People with Disability by scholars at the University of New South Wales, Sydney in Australia (Strnadová, Dowse & Garcia-Lee 2022). The guidelines’ six stages, namely *Initiating, Planning, Doing, Sense-making, Sharing* and *Reflecting*, are a promising tool in guiding research that includes people with ID as co-researchers that can be adapted for use in different contexts. Another recent milestone in this area was the launch of the International Association for the Scientific Study of Intellectual and Developmental Disabilities (IASSIDD)^1^ Special Interest Research Group (SIRG) on Inclusive Research on 16 December 2021 (for details, see https://iassidd.org/sirgs/inclusive-research/). The launch was followed by the SIRG’s first webinar on 22 March 2022 (for details, see https://www.youtube.com/watch?v=edKp5fFic2E). Within these developments, the author observed a lack of representation of African ID researchers on the global inclusive research platforms and a clear gap in literature on inclusive research from Africa as a continent. It is against this backdrop, that this article makes a call to African ID researchers to be part of the global developments in inclusive research. African researchers are encouraged to learn from the experiences of those using inclusive approaches and adopt the lessons as suitable to African contexts.

## Inclusive research in Africa

Capri and Coetzee (2012) published an enlightening opinion article on the ‘unethicality’ of excluding people with ID in research. They based their arguments on the human rights framework specifically citing exclusion of people with ID in research based on perceived cognitive incompetence as contravening the rights enshrined in the Constitution of the Republic of South Africa (*Constitution of the Republic of South Africa* [*RSA*], *No. 108 of 1996*). They further argued that the exclusionary practices contravene the social model of disability because the exclusion of people with ID based on the perceived limitations posed by their cognitive impairments is tantamount to disabling them. An expanded discussion of benefits of including people with ID in research and the risks and dangers of excluding people with ID or coercing them to participate in research is presented in their article. Ten years after Capri and Coetzee’s publication, research that engages people with ID is still scarce in South Africa and Africa at large. Although their article focused more on the South African context, the gap is arguably even more evident in the rest of Africa.

Although there have been some attempts to give a voice to people with ID in research in Africa, they are usually involved as participants (e.g. Ali et al. 2015; Bukhala et al. 2017) without any opportunities to influence what is researched and how the research is conducted. In other studies, issues concerning people with ID are explored by eliciting perspectives of family members or service providers of people with ID (e.g. Kahonde 2022; Malapela, Thupayagale-Tshweneagae & Mashalla 2020). There is also a sizeable number of publications focusing on professional practice, service delivery and policy (e.g. Kleintjes et al. 2020; Okyere et al. 2019). The common practice of conducting research *for* rather than *with* people with ID ‘further incapacitates already subdued voices’ as argued by Capri and Coetzee (2012:2).

As a continent, Africa is still lagging in recognising people with ID themselves as agents with autonomy to speak about their own lives and to get their voices heard in and through research as is the case in high-income contexts cited earlier. Related to this is the lack of self-advocacy by people with ID in Africa, yet self-advocacy skills are prerequisite for people with ID to engage in inclusive research effectively and successfully as concluded by Bigby et al. (2014) based on their review of literature on inclusive research. On the other hand, participating in inclusive research has the potential to empower people with ID with advocacy skills. With increased self-determination and agency, they can be emancipated from the paternalistic practices of professionals and family members who may have the tendency to want to speak on behalf of the person with ID (Chinn 2014). Their self-advocacy can also change stigmatising attitudes towards people with ID and erase their own self-stigma as their contributions to society are recognised and appreciated (Goldberg & Kleintjes 2022; Roth, Barak & Peretz 2016). Stigma towards people with ID is evident globally (Scior et al. 2020) but is arguably more prevalent in Africa because of poverty, low literacy levels, a lack of advocacy and services affirming the abilities of people with ID and sometimes spiritual explanatory models of ID (McConkey, Kahonde & McKenzie 2016; Mkabile & Swartz 2020).

## Challenges and obstacles to conducting inclusive research

It is imperative in this article to highlight the reasons identified in the literature as hindrances to the conducting of research that includes people with ID, which affect researchers internationally, albeit the literature discussing these challenges is mostly from high-income countries. Generally, research with people with ID whether they are participants or co-researchers is fraught with ethical challenges (Carlson 2013; Capri & Coetzee 2012; Iacono 2006). By its nature, ID may render the people living with this disability vulnerable to different forms of abuses and dangers, therefore, ethics review committees tend to apply more stringent measures to research that involves people with ID (Ramcharan 2006). It is common for researchers to be impacted by ethics review committees’ conundrum of desiring to protect people with ID while granting them the autonomy to choose whether they want to participate or not. Thus, the process of seeking ethics approval can become a daunting, back-and-forth process, sometimes requiring multiple levels of review and engagements with ethics review committees on the researcher’s part (Martino & Schormans 2018).

The paradox of coercion versus exclusion is an ongoing subject of debate and conflict (Carlson 2013). Iacono (2006:1) argued that the efforts to protect may inadvertently lead to ‘paternalistic protectionism, with a concomitant risk of non-inclusive and discriminatory decisions by institutional ethics committees’. Carlson (2013:305) calls this the ‘double danger of inclusion or exclusion’. This means that researchers may be faced with the dilemma of considering people with ID as vulnerable and in need of protection while the special considerations may consequently lead to their exclusion from research activities and opportunities for their voices to be heard and represented in research. From a human rights perspective, perceived vulnerability and assumption of homogeneity should not be a reason to exclude people with ID from research (Capri & Coetzee 2012; Martino & Schormans 2018). The increasing number of projects successfully and effectively engaging people with ID as participants or even more inclusively as co-researchers are evidence to prove such assumptions erroneous.

There is also gatekeeping by formal and family caregivers around the participation of people with ID, which is linked to medicalisation of ID and infantilisation of people living with this disability whereby caregivers view them as perpetual children needing protection (Martino & Schormans 2018). In some instances, researchers can get a research proposal approved by institutional ethics review committees but still have diminished chances of recruiting participants or co-researchers because of the gatekeeping rules of service providers and caregivers (Iacono 2006). In sum, inclusive research requires increased investment in time and resources on the part of the researcher, which relate to the ethics approval process discussed earlier, training of people with ID involved, cyclical data collection process that might require extra audio-visual equipment, for example, to make details of research accessible and understandable to people with ID and negotiating access to participants. Research grants may not cover these extra costs, and it adds greatly to the workload of doctoral students and other novice researchers (Martino & Schormans 2018). Literature is lacking from Africa explicating the challenges of conducting research with people with ID, neither is there literature documenting experiences of ID researchers and lessons learnt, which can inform and guide ID inclusive research on the continent.

## Promoting inclusive research in Africa

It has already been made clear that although inclusive research has been gaining ground among ID researchers in high-income countries for more than two decades, researchers in Africa are not prioritising inclusion of people with ID in research, particularly as co-researchers. This could be linked, among other factors, to the findings by Mckenzie, McConkey and Adnams (2014) in South Africa, that there is prioritisation of healthcare and protection for people with ID while giving little attention to autonomy and choice. More effort and priority need to be put in promoting and advocating for all rights of people with ID including the right to contribute meaningfully to research that seeks evidence to improve their lives.

As encouraged by Capri and Coetzee (2012), ID researchers in Africa are called to acknowledge and interrogate their own ignorance and a lack of knowledge when it comes to inclusion of people with ID in research. The same applies to the researchers’ ethical and moral values that may impact how they conduct the research and what they find (Capri & Coetzee 2012). As human beings, researchers are not immune to the negative stigmatising attitudes towards people with ID alluded to earlier. Combating stigmatising attitudes towards people with ID by promoting their visibility in the community through access to schooling and productive employment (McConkey et al. 2016; Scior et al. 2020) is critical. Giving them space for self-advocacy through social media, written content, campaigns and awareness-raising initiatives is also crucial (Goldberg & Kleintjes 2022). Organisations supporting people with ID also need to extend their advocacy to academic institutions and offer the support needed by researchers willing to co-research with people with ID. That way, academic researchers in Africa and the society at large can begin to appreciate the agency and capabilities of people with ID.

The required training of co-researchers with ID and the back-and-forth nature of inclusive research methods make the process resource intensive (Martino & Schormans 2018). Hence, the lack of resources is a likely deterrent for African researchers to undertake inclusive research. This is an area that needs to be prioritised by funders supporting African ID researchers, including the governments as it would be exclusionary and ableist to dismiss inclusive research because of financial reasons. The lack of resources might also explain the lack of representation of African researchers on platforms such as IASSIDD mentioned earlier, which normally hold their conferences in high-income countries, except for the virtual conferences necessitated by the COVID-19 pandemic. The current convenient and easily accessible virtual events and proceedings afforded by the COVID-19 pandemic restrictions are an opportunity that can be leveraged by African researchers to learn from those with experience while considering how to implement the lessons in their own unique contexts. Published inclusive research articles from other contexts also provide lessons for African scholars to learn from.

Although resources to train both people with ID and academic researchers in Africa may be scarce, the transformation must start somewhere. There is a need for innovative methods that are accessible to most people with ID in Africa, using inclusive research approaches that are relevant and responsive to the needs of the local context. One way to initiate inclusion with limited resources will be to adapt research methods and making data collection methods accessible to people with ID as much as possible, for example, through use of accessible data collection methods such as easy-to-read documents, social stories, pictures and other visual tools. A few researchers in Africa have attempted to do this (Balton et al. 2022; Kahonde & Johns 2022; Okyere, Aldersey & Lysaght 2021). Once people with ID get used to having their voices heard in research through accessible data collection methods, they may gain the skills and competence to engage academic researchers in studies that are more inclusive.

Crucially, training African researchers on inclusive research is imperative, and North–South and South–South collaborations (Boshoff 2010) are needed for researchers to learn from each other for the benefit of people with ID. Boshoff (2010) argued that these collaborations are critical for giving voice to researchers from the Global South who are usually underrepresented on global research platforms that are dominated by researchers from the Global North. Furthermore, South–South collaborations have an advantage of ‘shared problems and shared solutions’ (Kerr-Muir, Lehasa & Zondervan 2017) among nations from Global South who share similar contextual factors. The training of academic researchers must be paralleled with training of self-advocates as self-advocacy skills have been found to be essential (Bigby et al. 2014) for people with ID to competently engage in research with academic researchers as already stated. Also, the scarcity of inclusive research in Africa calls for training and education of ethics review committee members on inclusive research methods and processes to improve their understanding and skills in reviewing inclusive research proposals and guiding researchers. The training must emphasise a shift from the paternalistic blanket view of people with ID as a homogenous vulnerable group (Martino & Schormans 2018) to understanding that they are heterogenous, unique individuals who can benefit from research skills training and meaningful inclusion in research processes.

## Conclusions

Inclusive research with people with ID that uses context-relevant approaches is imperative in Africa. One finds evidence of research and documentation on research conducted by people with physical and sensory impairments as lead researchers (see e.g. Rohleder et al. 2021; Rule & Modipa 2012), but such type of research by people with ID is hard to come by. When the research is not inclusive of the perspectives of people with ID on what they regard as important to them, support services for people with ID may only address their needs as perceived by others and fail to meet their *actual* needs and rights. This can result in poor physical and mental health stemming from exclusion from health services, employment, leisure and community life in general. Inclusive research is also important for promoting advocacy skills among people with ID as a means of shifting from a paternalistic culture to an empowering culture, and this can further reduce the stigma commonly experienced by this group. This approach to research requires looking outside the existing systems and finding common cause with disability organisations of and for people with ID and for people with disabilities in general. It is hoped that this article and the global trends mentioned earlier can raise the interest of African researchers and ID support organisations in promoting and conducting inclusive research. Researchers will have to enter the terrain of inclusive research knowing the potential challenges but at the same time realising the immense benefits of inclusive research for people with ID and their communities.

## Footnotes

1 International Association for the Scientific Study of Intellectual and Developmental Disabilities is the biggest international organisation for intellectual disability researchers globally, which has membership from all continents of the world. It has the aim of promoting worldwide research and knowledge sharing and building of collaborations among researchers working on ID research (Timmons 2013).
